# Research Progress in Fluid Energy Collection Based on Friction Nanogenerators

**DOI:** 10.3390/mi15010040

**Published:** 2023-12-24

**Authors:** Jin Yan, Yuxuan Sheng, Dapeng Zhang, Zhi Tang

**Affiliations:** 1 Naval Architecture and Shipping College, Guangdong Ocean University, Zhanjiang 524088, China; 2 Guangdong Provincial Key Laboratory of Intelligent Equipment for South China Sea Marine Ranching, Guangdong Ocean University, Zhanjiang 524088, China; 3 Shenzhen Research Institute, Guangdong Ocean University, Shenzhen 518120, China

**Keywords:** triboelectric nanogenerator, fluid energy, collection device, optimized design, self-powered systems

## Abstract

In recent decades, the development of electronic technology has provided opportunities for the Internet of Things, biomedicine, and energy harvesting. One of the challenges of the Internet of Things in the electrification era is energy supply. Centralized energy supply has been tested over hundreds of years of history, and its advantages such as ideal output power and stable performance are obvious, but it cannot meet the specific needs of the Internet of Things, and distributed energy supply also has a large demand. Since the invention of nanogenerators, another promising solution for fluid energy harvesting has been opened up. The triboelectric nanogenerator is an emerging platform technology for electromechanical energy conversion, which can realize the collection of fluid energy such as wind energy and wave energy. In this paper, we first introduce the fundamentals of triboelectric nanogenerators and their applications in wind and wave energy harvesting devices. We then discuss the methods of device optimization in the next development of TENG and conclude by considering the future prospects and challenges for triboelectric nanogenerator harvesting devices.

## 1. Introduction

The evolution of energy streaks across the sky has been lighting up the dawn of technological development. The price of chemical energy has gradually deviated from the ideal acceptable range, energy usage has increased sharply every day, and collecting environmental energy has been favored by researchers. It is also extremely difficult to build and maintain a certain amount of power plants in countries and regions with high construction costs, high site selection requirements, large maintenance and labor needs, backward living standards, or complex geographical environments [1,2,3,4]. Entering the 21st century, the Internet of Things has achieved milestone development. Whether it is medical health, daily life peripherals, or even industrial production and transportation, there are a large number of sensors, and the power gap is only increasing. The distinctive feature of this type of power demand is that it is not physically located and spreads across various spaces. Assuming battery power is used, the pollution to the environment will be immeasurable. Frequent replacement of batteries is likely to result in uncontrollable front-end and post-production costs and maintenance costs. In order to solve these problems, a large number of green energy supply devices have been developed, such as solar photoelectric converters [5,6], wind turbines [7], thermoelectric generators [8,9], fuel cells, etc. Solar converters require a large initial investment, occupy a large area, and have slow cost recovery. Wind turbines are similar to the former, but more attention should be paid to safety and noise issues. Throughout human history, the triboelectric effect has been seen as a detrimental occurrence in science and technology. Uncontrolled high-voltage static electricity consumes energy and increases the danger of electromagnetic interference and electronic component failure. It must be minimized, if not completely protected. There are several motion modes, including heave, rotation, and vibration, while gathering fluid kinetic energy. These energies have high entropy, low frequency, and chaos. 

In the long river of human exploration of science and technology, the frictional electrification effect has long been regarded as a negative phenomenon. Uncontrolled high-voltage static electricity not only carries the risk of electromagnetic interference and breakdown of electronic components but also wastes energy, which needs to be reduced or even shielded as much as possible. In terms of fluid kinetic energy collection, there are motion modes such as vibration, oscillation, and rotation, which are characterized by disorder, low frequency, and high entropy. If these unnecessary high entropy fluid energies are collected using Faraday electromagnetic induction generators (EMGs) based on the law of electromagnetic induction, some EMGs need to operate under certain high-frequency motion, and some also need to exhibit certain motion trends, such as the certainty of rotation direction. Relatively speaking, the high entropy characteristics of the fluid capacity collection can lead to a significant waste of inexhaustible low-frequency vibration energy, including wave energy and wind energy. In 2012, Wang Zhonglin’s team proposed a conversion device that utilizes the electrostatic effect, triboelectric nanogenerators [10]. In terms of cost, since the human energy revolution entered the era of electrification, most of the sources of electricity obtained by humans have come from electromagnetic induction generators (EMGs). EMG is equipped with a large number of iron cores and copper coils inside, which is costly. Unlike EMG, nanogenerators use a large amount of polymer materials, which are easy to obtain and have low manufacturing costs. In the operation of multiple single-unit parallel networking, multiple nanofriction generators can be assembled into a large-scale power storage network through specific control and energy storage circuits. The light weight, the fluid energy harvesting response efficiency in low-frequency, low amplitude, and high entropy vibrations are much better than that of electromagnetic induction generators, making nanogenerators an ideal energy harvesting component for the new generation.

Under low-cycle conditions, the energy conversion efficiency of nanogenerators is much higher than that of electromagnetic induction generators suitable for high-cycle cycles. An energy conversion device converts mechanical energy into thermal energy or other energy when mechanical energy with a low circulating frequency is expressed in the form of pulses. For example, shock absorbers convert mechanical energy into thermal energy by setting damping. The development of clean energy and the conversion of the remaining mechanical energy into other energy can not only extend the service life of the equipment and reduce the maintenance cost but also improve the energy utilization rate by using energy reserves. The theoretical core of nanogenerators is Maxwell’s equations. Maxwell’s equations are as follows. The first term is the induced current caused by changes in the electric field, which provides a prerequisite for wireless communication. The second term is the displacement current caused by the polarization field generated by the electrostatic charge carried on the surface of the medium, which becomes the theoretical basis of the triboelectric nanogenerator [11,12,13].

(1) $$J_{D}=\frac{\partial D}{\partial t}=\varepsilon \frac{\partial E}{\partial t}+\frac{\partial P_{s}}{\partial t}$$

where *D* is the displacement field, *E* is the electric field, *t* is the time, *ε*_0_ is the vacuum permittivity, and *P_s_* is the current caused by the polarization field caused by the charge on the surface of the dielectric.

**Figure 1:**
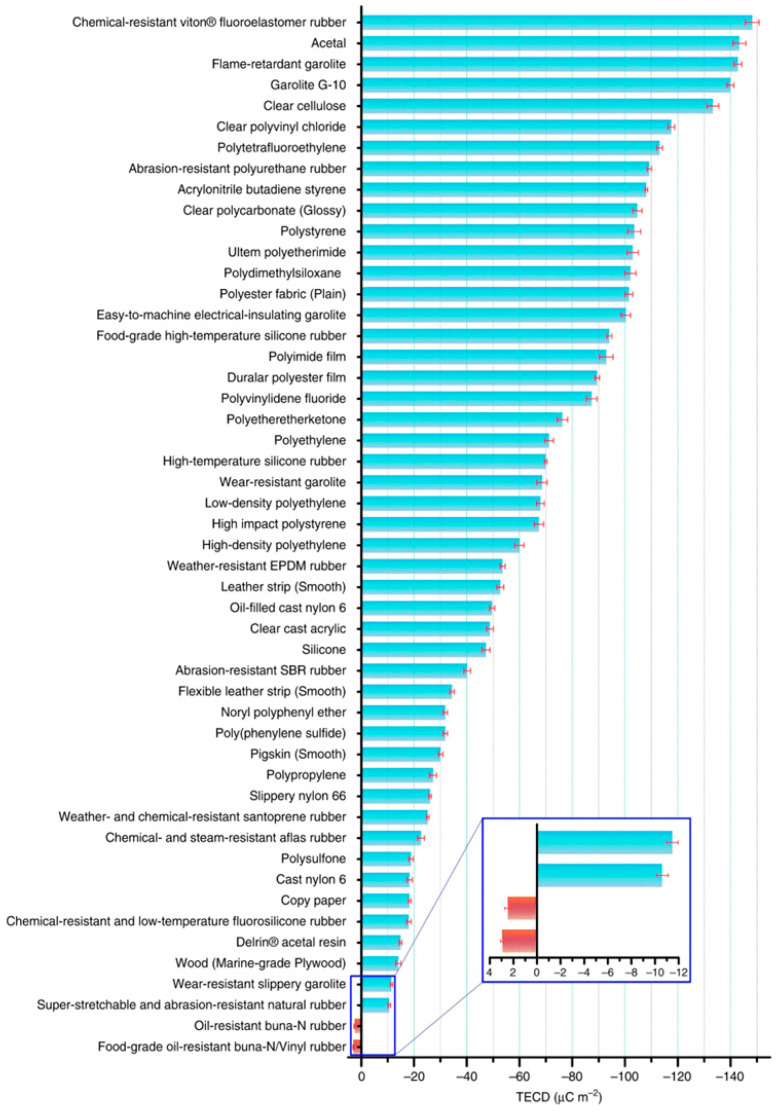
Triboelectric sequences of commonly used friction layer interface materials. Reproduced with permission from Ref. [13]. Copyright, 2019, The Author(s). Published by Nature Communications.

At present, electrodes and triboelectric layers are required components of all commercially available triboelectric nanogenerator materials. Using the vertical contact-separation mode as an example, charge communication between the nanogenerator and the external circuit load is facilitated by two metal electrodes with low internal resistance located on the outermost side. Two metal electrode pieces are encased in two pieces of a certain substance. The triboelectric series contains these two materials. Figure 1 displays the triboelectric sequence of widely used triboelectric materials. Generally, in order to increase the power density of power generation, it is necessary to look for materials near both ends of the triboelectric sequence, aiming to find materials that tend to be “positive” and tend to be “negative”. The “tendency” here indicates that the attraction of materials to electrons is different, with materials that tend to be “positive” being more likely to lose electrons, and materials that tend to be “negative” are more likely to gain electrons [14,15].

**Figure 2:**
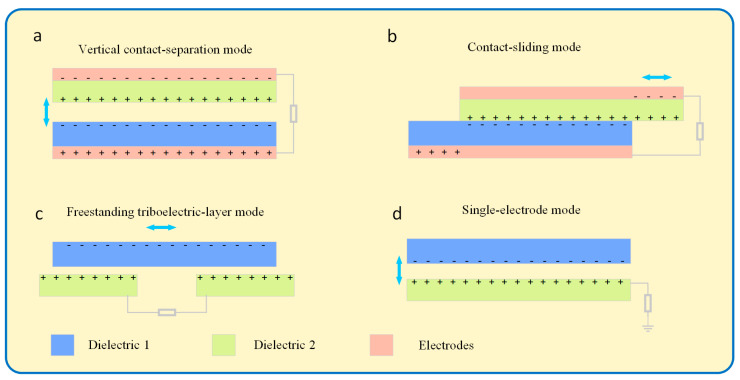
Currently, there are four main modes of research on nanogenerators. (**a**) Vertical contact separation mode, (**b**) lateral sliding mode, (**c**) single electrode mode, (**d**) independent frictional layer mode.

Solid–solid coupling nanogenerators are now the subject of further in-depth investigation. Figure 2 depicts the single electrode mode, independent layer mode, vertical contact-separation mode, and horizontal sliding mode of solid–solid coupling nanogenerators. According to various purposes, numerous fundamental modes may be employed [16,17]. According to the triboelectric sequence, polyimide (Kapton) tends to gain electrons from polymethacrylic acid (PMMA). As the friction layers approach or recede from one another, the air gap in the friction layer continues to diminish, causing a charge to move and a potential difference to form. A significant component of current research endeavors involves horizontal slip nanogenerators. To finish the power production process, two triboelectric material pieces with electrodes connected on the rear slide parallel to the joint surface may be made to engage in friction or there may be a gap between the friction layers that results in relative displacement [18]. Figure 2c depicts the single-electrode mode nanogenerator. As a zero-potential reference point, it has a reference electrode. When propelled by an external force, the moveable electrode produces a potential difference or current, much like the vertical contact-separation or sliding mode. The electrified friction layer is liberated from the confines of the wire, which is a significant benefit [19]. The structure of the independent-layer modal nanogenerator is shown in Figure 2d, which is similar to the horizontal slip mode, and the friction layer can be designed to be in contact and non-contact state.

## 2. Recent Developments in Triboelectric Nanogenerator-Based Fluid Energy Harvesting Systems

The mechanical characteristics of friction nanogenerators determine their suitability for application in the field of low-frequency periodic collection. Most fluids in nature have lower vibration periods, providing development space for nanogenerators. This article will briefly introduce the use of green and environmentally friendly nanofriction generators from the following two aspects: the first is the theoretical and material research on nanofriction generators. The second is about the collection of low-frequency energy from nanogenerators, including wave energy, wind energy, etc., which have broad application prospects in green and blue energy. Under low cycle conditions, the energy conversion efficiency of nanogenerators is much higher than that of electromagnetic induction generators suitable for high cycle conditions. When mechanical energy with lower cycle frequencies is expressed in pulsating form, an energy converter converts mechanical energy into thermal energy or other energy. For example, shock absorbers convert mechanical energy into thermal energy by setting damping. Developing clean energy and converting surplus mechanical energy into other forms of energy can not only extend the service life of equipment and reduce maintenance costs but also reserve energy and improve energy utilization efficiency [20].

### 2.1. Wind Energy Collection

Wind energy has the benefit of sustainable supply, as is well known as being a clean energy source. The electromagnetic induction generators and turbines seen in modern wind power plants are advantageous due to their big power production and reliable operation [21]. The installation site is very demanding, and the electromagnetic induction generator performs poorly at low speeds, thus a more complicated ramp-up mechanism is necessary, which is an inherent disadvantage [22,23]. The high-speed rotation of the propeller or the movement of the large-size propeller causes noise problems that can only be solved by optimization methods [24,25]. The simple structure and low cost of nanogenerators can complement traditional wind power generation. The composition of nanogenerators also has the advantage of being green and environmentally friendly, and organic or inorganic materials that are easily absorbed and degraded by the environment and minimize dependence on precious metals can be selected, and environmentally friendly friction layer materials can be continuously developed [26,27,28,29]. However, the collection of wind energy is difficult, especially at lower wind speeds, which has not been widely covered in current research, and with the flow of air currents, a large amount of energy is not collected and wasted, as a result, the utilization rate of wind energy is still not high [30]. In this section, we will discuss the topologies of wind nanogenerators and divide them into rotating structures, flutter structures, and other structures that are not easy to classify.

#### 2.1.1. Rotating Structure AC Output Device

**Figure 3:**
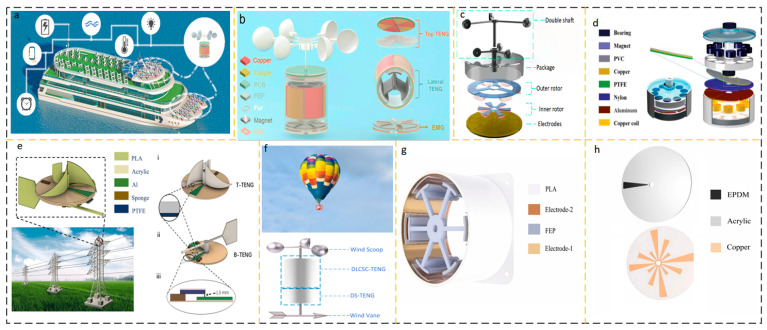
AC output device based on rotating structure. (**a**) RS-HG application scenario and (**b**) RS-HG structural design. Reprinted with permission from Ref. [31]. Copyright 2023, Wiley-VCH GmbH. (**c**) D-TENG structural design. Reprinted with permission from Ref. [32]. Copyright 2021, Wiley-VCH GmbH. (**d**) The hybrid generator structural design. Reprinted with permission from Ref. [33]. Copyright 2021, Wiley-VCH GmbH. (**e**) WM-TENG application scenarios and structural design. (i) T-TENG is used for wind speed measurement, (ii) B-TENG is used for wind direction monitoring. (iii) side view showing the sensing distance between PTFE and Al. Reprinted with permission from Ref. [34]. Copyright 2022, Elsevier. (**f**) MM-TENG application scenarios and structural design (**g**) DLCSC-TENG structure and (**h**) DS-TENG structural design. Reprinted with permission from Ref. [35]. Copyright 2023, Elsevier.

The conventional meteorological wind speed sensor assembly achieves high flexibility by using several revolving wind cups and rotational sensor architectures. Utilizing high-efficiency impellers or wind cups for the rotational drive, nanogenerator energy harvesting devices are often used in low-wind situations. This conventional rotating construction could deliver outstanding energy production. Cao et al. proposed a high-performance triboelectric-electromagnetic hybrid nanogenerator (RS-HG) with a rotating structure [31]. The application setup is shown in Figure 3a, the structure is shown in Figure 3b, and the RS-HG structure consists of transverse TENG (L-TENG), top TENG (T-TENG), and bottom electromagnetic generator (EMG). Increasing the bandwidth of the collected wind velocity increases the application of TENG. As shown in Figure 3c, Shun Yong et al. reported a coaxial structure with a dual-axis D-TENG. The D-TENG has two cups with different shapes and arm lengths, and the two different cups are mounted on a coaxial shaft to complement each other’s rotational characteristics and harvest energy at ultra-wideband wind speeds of 2.2–16 m/s [32].

Zhao et al. reported a wind energy harvesting device that combines electromagnetic induction generator EMG and sliding friction nanogenerator TENG with the structure shown in Figure 3d [33]. When the wind speed is 9 m/s, the maximum average power outputs that can be achieved are 0.33 and 32.87 mW, respectively. Overhead power lines are often disturbed by wind direction, posing a serious potential risk factor. As shown in Figure 3e, Tang et al. proposed a non-contact WM-TENG, which consists of T-TENG and B-TENG. The T-TENG is driven by a turbine, while the B-TENG located at the bottom is driven by a wind vane [34]. The wind sensor on the hot air balloon is an important part of the hot air balloon safety system. Traditional wind sensors on hot-air balloons are powered by lithium batteries, which have unsatisfactory reliability. Gu et al. proposed a self-powered positioning system for hot air balloons based on a multi-module triboelectric nanogenerator (MM-TENG) as shown in Figure 3f. The structure is cylindrical, with a double-layer cylindrical soft-contact triboelectric nanogenerator (DLCSC-TENG) on the side of the cylinder (Figure 3g) and a disc-shaped triboelectric nanogenerator (DS-TENG) on the underside of the cylinder (Figure 3h). The wind speed threshold of DLCSC-TENG is reduced to 0~6 m/s, and eight regular directions can be monitored through the output voltage waveform [35]. The main theme of these studies is focused on using wind cups or blades with sufficient strength and moment of inertia, and a certain direction of incident airflow to drive the rotor of energy harvesting equipment, driving the friction interface layer and generating AC current output of traditional TENGs. Table 1 summarizes the performance of some wind energy collection AC output devices in recent years.

#### 2.1.2. Rotary Structure DC Output Device

**Figure 4:**
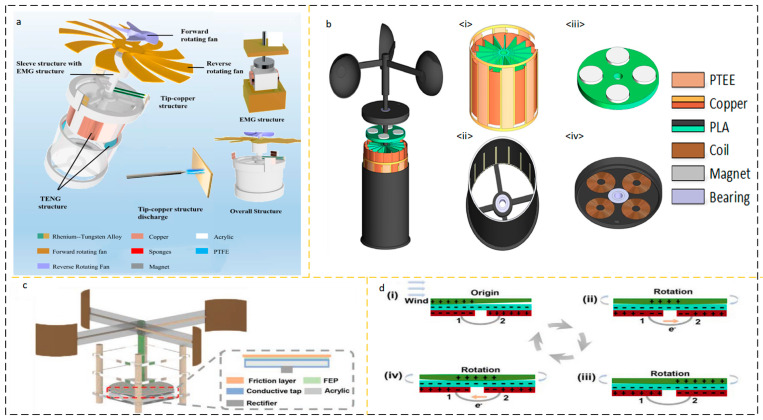
A DC output device based on a rotating structure. (**a**) TENG-EMG harvesting using a hybrid energy combination of tip discharge and TEMG and EMG integration. Reprinted with permission from Ref. [36]. Copyright 2023, Elsevier. (**b**) WH-EH device with integrated soft friction SP-TENG and layered rotating HR-EMG, (i,ii)TENG structure, (iii,iv) EMG structure. Reprinted with permission from Ref. [37]. Copyright 2022, Science China Press and Springer–Verlag GmbH Germany, part of Springer Nature. (**c**) Schematic diagram of the working principle of WNG consisting of a circular wedge-shaped face and a disc. Reprinted with permission from Ref. [38]. Copyright 2022, Wiley-VCH GmbH. (**d**) Schematic diagram of working principle: (i) initial state; (ii) The friction interface begins to rub and generate charge transfer; (iii) Continue friction; (iv) Generate charges in the opposite direction.

In the traditional TENG, the output is alternating current in the direction of change, but in order to achieve reasonable energy storage and energy use, it is likely to add rectifier current to convert AC into DC, and then convert it into the required electrical energy through buck-boost and voltage stabilization circuits. Juan Cui et al. proposed a TENG-EMG, as shown in Figure 4a. Similarly, TENG and EMG are integrated. In terms of EMG, the flipping magnet is combined with the coil to improve the magnetic flux change of the cutting coil. In terms of TENG, the output current uses charge accumulation during rotation through the sidewall tip copper structure to achieve gas ionization and increase [36]. 

A wind energy hybrid harvester (WH-EH) combining soft friction forward triboelectric nanogenerator (SP-Teng) and hierarchical rotating electromagnetic nanogenerator (HR-EMG) was proposed by Yuan et al. As shown in Figure 4b, in the columnar structure, the HR-EMG is located at the bottom of the column, and the SP-TENG is on the side of the column, designed by combining electrodes and inner wall beams [37]. An ultralow friction and efficient pinwheel-shaped nanogenerator (WNG) by Zhu et al., WNG uses a rotating triboelectric layer with contact-separation mode. As shown in Figure 4c,d, WNG is composed of a circular wedge-shaped plane turntable. When the windmill drives the circular wedge-shaped surface to rotate, the wedge-shaped surface and the disc continue to move closer to reach the contact-separation mode to generate electricity. The contact between the circular wedge surface and the disc reduces the friction resistance between the friction pairs and reduces the threshold of WNG starting wind speed. The optimized WNG maximum load power is 0.753 μW, which can light up nine LEDs at the same time [38]. Introducing magnets and coils or cleverly designing wedge-shaped contact surfaces to control the polarity of charge output without changing, achieving DC pulse output. Table 2 summarizes the performance of some wind energy collection DC output devices in recent years.

#### 2.1.3. Rotating Self-Adjusting Structure Output Device

**Figure 5:**
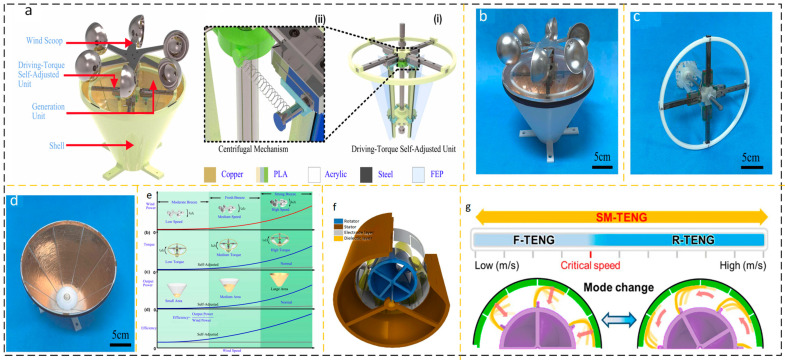
Output device based on a rotating self-adjusting structure. (**a**) (i) SA-TENG structural design (ii) Self-adjusting torque structure. (**b**–**d**) Digital image of each part of SA-TENG. (**b**) photos of of Self-adjusting torque structure, (**c**) photos of SA-TENG rotor, (**d**) photos of SA-TENG stator. (**e**) SA-TENG working mode switching under different wind speeds. Reprinted with permission from Ref. [39]. Copyright 2022, Elsevier. (**f**) SM-TENG structure and (**g**) SM-TENG sliding and contact separation mode switching principle. Reprinted with permission from Ref. [40]. Copyright 2023, Elsevier.

The wind speed acquired through the utilization of a standard rotating mechanism energy harvesting device may not exhibit consistent stability. To address this issue, a strategy is implemented wherein multiple sets of structures are arranged in parallel. Each set of structures is designed to respond to different wind speed intervals. Additionally, another approach involves enhancing the efficiency of the device’s response by optimizing the geometry of its structure. However, the performance of the aforementioned mechanisms in capturing wind speed response is still limited. Wang et al. reported a triboelectric nanogenerator (TENG) with a self-adjusting driving torque, as shown in Figure 5a(i). This design was motivated by the feedback structure inherent in the mechanical system. The transition between contact and separation modes is contingent upon the wind speed input. The wind cup and the rotating structure are interconnected with the torque self-adjusting unit, as seen in Figure 5a(ii). The power-generating surface is designed in a trapezoidal form, as seen in Figure 5d. Under the action of different wind speeds, the centrifugal angle of PLA changes with the change of wind speed, resulting in inconsistent contact area with the trapezoidal copper power generation surface to match the input of different wind speeds, as shown in Figure 5e [39]. Sumin Cho proposed an SM-Teng that adjusts the working mode according to different wind speeds, as shown in Figure 5f. SM-TENG switches between contact and separation modes according to the input wind speed. As shown in Figure 5g, when the wind speed is less than 5 m/s, the working mode of SM-TENG is sliding F-TENG. When the wind speed exceeds 5 m/s, SM-TENG works in contact with the rotating separated R-Teng [40]. Changing the rotational inertia or driving torque of the device, as well as changing the key characteristics of the mechanism itself at different wind speeds, such as changing the contact area of the friction layer due to the centrifugal angle, achieves good characteristics of the mechanism’s self-adjustment output with wind speed changes.

Table 3 summarizes the performance of some self-adjusting wind power generation devices based on rotating structure in recent years.

#### 2.1.4. Energy Harvesting Device Based on Flutter Structure

**Figure 6:**
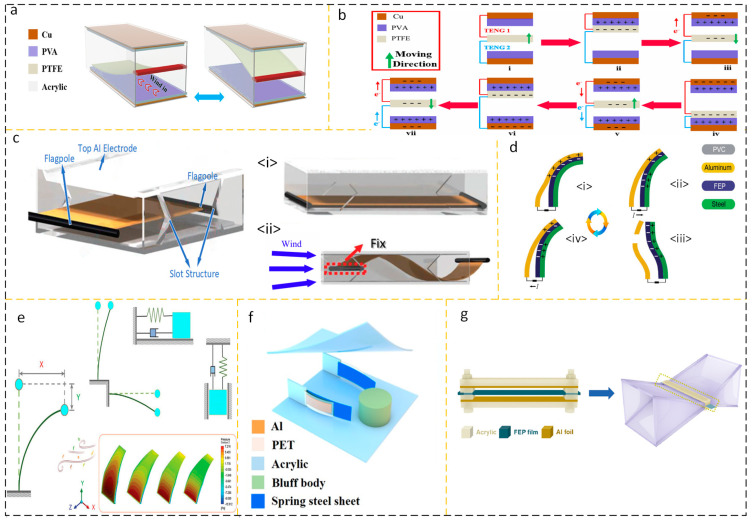
(**a**) PTFE-based flexible wind-driven TENG and (**b**) working cycle diagram. (i) Under the influence of airflow, PTFE flutter blade moves upward; (ii) the PTFE sheet contacts the upper plate, and the electrons in PVA are transferred to PTFE due to their electronegativity differences, resulting in positive charges on the PVA surface and negative charges on the PTFE surface; (iii) the potential difference between the top copper electrodes can drive the negative friction charge from PTFE to the copper electrode surface; (iv) similarly, due to the influence of air flow, PTFE approaches the lower PVA film and generates a positive charge on the PVA surface, while PTFE generates a negative charge on the surface; (v) When the middle friction layer of PTFE moves upward, the negative friction charge will be transferred from PTFE to the copper electrode surface at the bottom of Teng, resulting in a potential difference between PTFE and the top copper surface, and driving the negative friction charge from the top copper surface to PTFE. Reprinted with permission from Ref. [41]. Copyright 2021, Elsevier. (vi) The PTFE vibration plate moves upwards until it comes into contact with PVA, and the electronegativity of the two materials is not consistent, resulting in contact charges; (vii) When PTFE vibrators leave PVA, the charge balance of an equal amount of contact charge is disrupted, relying on the external circuit to transport the charge, causing the negative charge in the external circuit to move. (**c**) (i) WBF-TENG structural diagram. (ii) When the air flow passes through the inlet, the blown film vibrates and generates friction charges due to the electronegativity difference of materials. (**d**) (i) Without airflow excitation, there are equivalent heterogeneous charges on the contact surfaces of aluminum and FEP; (ii) when there is airflow excitation and aluminum and FEP are separated, the difference of electronegativity causes the friction charge of aluminum to flow to the steel; (iii) aluminum is completely separated from FEP, and all charges are transferred to steel; (iv) aluminum is in contact with FEP, and the charge of steel is transferred back to aluminum.Reprinted with permission from Ref. [42]. Copyright 2022, Wiley-VCH GmbH. (**e**,**f**) Flutter working principle of LL-TENG. Reprinted with permission from Ref. [43]. Copyright 2023, Wiley-VCH GmbH. (**g**) Realizing the WG-TENG structure using wake propagation. Reprinted with permission from Ref. [44]. Copyright 2022, Elsevier. (**g**) W-TENG structure (after adding channels) that collects weak airflow by adding a shroud. Reprinted with permission from Ref. [45]. Copyright 2022, Elsevier.

The effect of humidity on nanogenerators in wind energy harvesting environments is a significant field of research. As a result, TENG’s output performance suffers and its service life is drastically lowered. Triboelectric charges were generated between a hydrophobic polytetrafluoroethylene (PTFE) flexible film and a polyvinyl alcohol (PVA) film, as shown by Sun et al. Figure 6a,b illustrate the design and operation of the device, respectively. At 95% RH (relative humidity), the optimized short-circuit current is 29.72 µA, the output voltage is 695.18 V, and the optimized power is 1.74 mW [41]. Different from previous studies, which focused on unilateral fixation of the windward side of the flutter film, Jin-ho Son et al. proposed a wind-driven bidirectional flutter triboelectric nanogenerator with bilateral fixation (WBF-TENG). The membrane fixed on both sides provides two stabilization lines, which can effectively receive airflow from more directions. Its structure is shown in Figure 6 [42]. 

In order to further reduce the wind speed response threshold of the flutter structure TENG, increasing the windward surface is a feasible solution. A triboelectric nanogenerator (TENG) with a response threshold of 0.2 m/s was suggested by Li et al., as illustrated in Figure 6d. As can be seen in Figure 6e [43], the flexible blades, vein-bearing plates, and counterweights that make up the foliate structure enhance the frontal area while also providing the generator with a precisely calibrated damping ratio. Yuan and colleagues created a wake-driven triboelectric nanogenerator (WG-TENG) based on the sweeping spoiler. The WG-TENG consists of two flutter plates and a spoiler column and shell in the center, as seen in Figure 6f [44]. Zhu et al. used the principle of aerodynamics to optimize the airway structure and designed a flutter structure nanogenerator with an inlet channel (W-TENG) to successfully gather the dispersed weak airflow flow energy, as shown in Figure 6g. The opening speed threshold of W-TENG is reduced to 0.4 m/s, and the average output voltage is 6.1 V [45]. Researchers have developed several efficient solutions to the problem of capturing erratic airflow.

**Figure 7:**
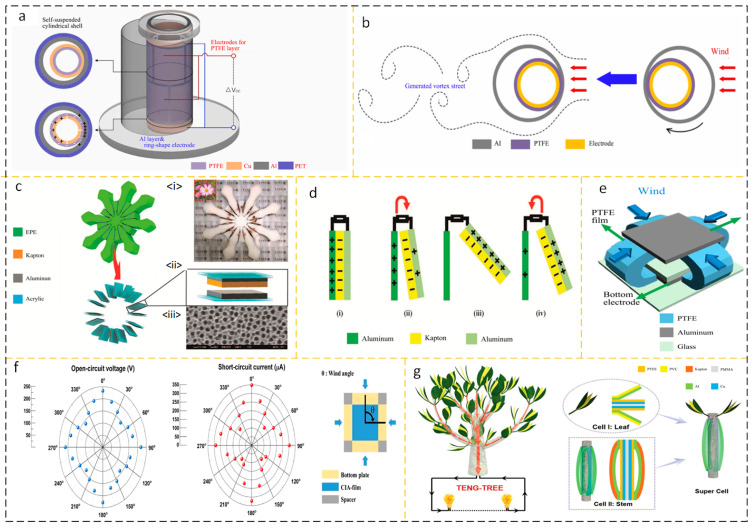
(**a**) Structural diagram of the self-suspended shell-based triboelectric nanogenerator and (**b**) working condition using the turbulent current of the shell. Reprinted with permission from Ref. [46]. Copyright 2022, Elsevier. (**c**) C-TENG structure, (i) C-TENG digital photo (ii) schematic diagram of friction layer and (iii) microstructure diagram. (**d**) The duty cycle of each blade. (i) In the absence of airflow excitation, there exists an equivalent non-uniform charge on the contact surface between aluminum and Kapton; (ii) When there is airflow excitation and aluminum and Kapton are separated, the charge of the left aluminum flows towards the right aluminum; (iii) Aluminum is completely separated from Kapton and all charges are transferred to the right aluminum; (iv) When the Kapton on the right approaches the aluminum on the left again, the charge transfers back to the aluminum sheet on the left. Reprinted with permission from Ref. [47]. Copyright 2022, the Authors. Published by Elsevier Ltd. (**e**) C-TENG and CIA-TENG structure and (**f**) good wind performance with multiple incidence angles. Reprinted with permission from Ref. [48]. Copyright 2021, the Author(s). (**g**) Application diagram and structure of a tree-structured triboelectric nanogenerator Reprinted with permission from Ref. [48]. Copyright 2018 WILEY-VCH Verlag GmbH & Co. KGaA, Weinheim. In recent years, the performance of some wind energy collection devices based on flutter structures has been summarized in Table 4.

Hee-Jin Ko et al. proposed a TENG composed of aluminum and polytetrafluoroethylene (PTFE). The nanogenerator consists of two hollow embedded cylindrical shells. The outer cylindrical shell is made of PET embedded with aluminum film, and the inner cylindrical shell is made of PTFE embedded with copper film. Its structure is shown in Figure 7a. Due to the Coulomb attraction between aluminum and PTFE, the self-suspended structure of the thin shell is formed. When the turbulent shell is disturbed at a certain wind speed, as shown in Figure 7b, the change in the relative distance between the two charged layers enables the energy collection element to pass through the triboelectric energy conversion mechanism. And thanks to the cylindrical structure, the incident directivity factor of the energy harvesting device does not need to be overly clear [46]. Inspired by a new type of caliopsis, Zhao et al. designed a TENG (C-TENG) that harvests energy or can be used as a self-powered sensor. When the frequency is 5 Hz and the wind speed is 154 m/s, the open circuit voltage (*Voc*) of a single C-TENG can reach 61.7 V and the short circuit current (*Isc*) can reach 0.341 A; when the load resistance is 100 MΩ, the peak value of a single C-TENG is reached. The output power is 11.57 mW/m^2^, as shown in Figure 7c,d, respectively, showing its structural design and working cycle [47]. Yoseop Shin proposed a TENG structure with a four-way curved dielectric film, as shown in Figure 7e. An aluminum layer is inserted into the dielectric film to improve the electrostatic induction, thereby improving the triboelectric performance, and good wind resistance can be seen in Figure 7f [48]. Inspired by the tree structure, Yaoxing Bian et al. designed a supercell tree consisting of leaf cells 1 and stem cells 2 as shown in Figure 7g. With an input wind speed of 11 m/s, it can produce a *Voc* of 330 V, an Isc of 59.6 μA, a matching resistance of 60 MΩ, and an output power of 3.6 mW [49]. In the design of TENGs for flutter structures, flutter plates have become a key factor in collecting wind speed thresholds for this energy harvester due to their inherent natural frequency and allowable swing. In addition, a reasonable air duct design can improve the response characteristics of this device, and the continuously decreasing wind speed threshold is its manifestation.

**Table 4:** Performance of wind energy harvesting devices based on flutter structure in recent years.

TENG	Operating Wind Speed Range	Short-Circuit Current	Open-Circuit Voltage	Power/Power Density	Ref.
PTFE-based flexible wind-driven TENG	10 m/s	29.72 μA	695.18 V	1.74 mW	[41]
WBF-TENG	3~4 m/s	6.9 μA	97.9 V	$1.98\;{mW/m}^{2}$	[42]
LL-TENG	0.2~2.5 m/s	7.9 μA	338 V	2 mW	[43]
WG-TENG	1~8.1 m/s	3.4 μA	175 V	$149\;{mW/m}^{2}$	[44]
W-TENG structure after adding channels	0.4~15 m/s		150 V	0.6 mW	[45]
Self-suspended shell-based triboelectric nanogenerator	0.3~10 m/s	119.8 V		$8.43\;{mW/m}^{2}$	[46]
C-TENG	15~17.1 m/s	0.341 A	61.7 V	$11.57\;{mW/m}^{2}$	[47]
C-TENG and CIA-TENG	3~9 m/s	348 μA	233 V	$46.1\;{mW/m}^{2}$	[48]
Tree-structured triboelectric nanogenerator	4~17 m/s	59.6 μA	330 V	3.6 mW	[49]

### 2.2. Wave-Based Energy Harvesting Device

Today, coal is still an important resource for people, and large amounts of pollution is an unavoidable problem in thermal power plants [50,51]. The research and practice of hydropower have a history of hundreds of years, and the application of hydropower has wide applicability [52]. The collection of ocean energy or other water flow energy has become a current research hot topic. The collection of ocean wave energy must take into account the corrosion problems of components caused by various ions in seawater [53,54,55]. Traditional ocean wave energy collection equipment has complex structures [56,57], high manufacturing process requirements, large size, and difficult maintenance. The wave energy in the low-frequency band contains a large amount of potential energy, but traditional energy collection devices cannot collect it, resulting in the dissipation of a large amount of energy [58]. Nanogenerators can collect low-frequency, instantaneous energy, which determines their potential to become a research hotspot [59]. This section will start with nanogenerators for water flow and wave energy collection applications, and briefly introduce the research progress of nanogenerators for water flow and wave energy collection through different composition structures.

#### 2.2.1. Cylindrical Structure AC Output Device

**Figure 8:**
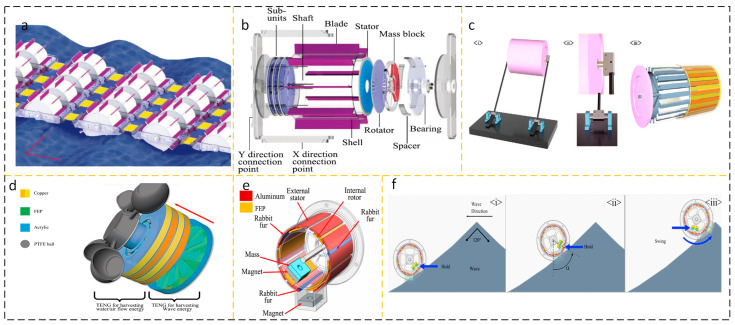
(**a**) WS-TENG Working Group Network and (**b**) individual WS-TENG diagrams. Reprinted with permission from Ref. [60]. Copyright 2022, Wiley-VCH GmbH. (**c**) (i) WLM-TENG (ii) junction of linkage-cylindrical structure and (iii) cylindrical TENG module. Reprinted with permission from Ref. [61]. Copyright 2022, Wiley-VCH GmbH. (**d**) Diagram of the structure of Murthion Ketionar’s pain. Reprinted with permission from Ref. [62]. Copyright 2017, WILEY-VCH Verlag GmbH & Co. KGaA, Weinheim. (**e**) Schematic diagram of a single FMC-TENG structure and (**f**) FMC-TENG motion in waves. (i) FMC-TENG initial time; (ii) FMC-TENG is subjected to wave excitation, and due to its own mass block, it undergoes rotational motion along the axis of the cylinder. (iii) The state when reaching the highest altitude. Reprinted with permission from Ref. [63]. Copyright 2022, Battelle Memorial Institute. Published by Elsevier Ltd.

In energy harvesting devices using water as the medium, the columnar structure is widely used. The collection of wave energy in ocean waves has the characteristics of low amplitude and randomness. The cylindrical structure is easy to adapt to this kind of random energy, its dynamic characteristics are excellent, and the stability is good. Hu et al. proposed a wheel-shaped triboelectric nanogenerator (WS-TENGs), the appearance of which is shown in Figure 8a. As shown in Figure 8b, the cylindrical structure of the WS-TENGs is equipped with an impeller on the cylinder and a TENG on the side, which is agitated by a wave to turn the TENG [60]. Han et al. (Figure 8c) proposed a TENG (WLM-TENG) with an *Isc* up to 30 μA unidirectional rotating wave-driven cylindrical linkage mechanism with an output power of 50 mW [61]. 

In the field of energy harvesting, a large number of studies on nanogenerators can only collect the energy of a single fluid medium, such as wind energy or wave energy, and it is rare to collect the energy of two fluids at the same time. Yi Xi et al. proposed three types of energy TENG (multifunctional TENG) that can be used to collect them, including water waves, air flow, and water flow, as shown in Figure 8d. When the wind blows the TENG to a rotation speed of 200 rpm, Voc reaches 490 V and Isc reaches 24 µA; 3 Hz; at a motion frequency of 100 V, *Isc* reaches 2.7 µA [62]. As shown in Figure 8e, Jung et al. used a magnet to store potential energy and immediately release it to generate a frequency-doubled cylindrical TENG (FMC-TENG) with high-frequency kinetic energy. As shown in Figure 8f, the bottom of the FMC-TENG is equipped with two permanent magnets. The mutually exclusive polarity between the permanent magnets is used to further store potential energy and increase the flipping ability of the internal rotor. When the excitation wave frequency is as low as 0.33 Hz, the peak power density reaches 6.67 mW/m^3^ [63]. In recent years, the performance of some AC output wave energy harvesting devices based on cylindrical structures has been summarized in Table 5.

#### 2.2.2. Cylindrical DC Output Device

**Figure 9:**
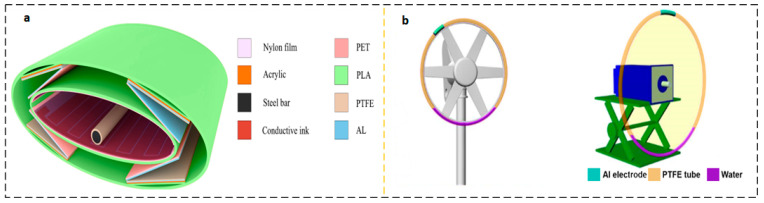
(**a**) EC-TENG structure. Reprinted with permission from Ref. [64]. Copyright 2022, the Authors. Published by Springer Nature. (**b**) TENG structure designed by Wang et al. Reprinted with permission from Ref. [65]. Copyright 2018, Wiley-VCH Verlag GmbH & Co. KGaA, Weinheim.

The power output of AC TENG is more difficult than that of DC output TENG in terms of direct utilization and energy storage, so it is necessary to develop DC output TENG. In addition, energy harvesting in harsh sea conditions is a gap in the current research on nanogenerators for wave energy harvesting, and it is important to ensure the structural stability of TENG at sea to prolong the life cycle of energy harvesting. Tan, D. et al. proposed an elliptical-cylindrical structure based on TENG (EC-TENG) with overturning resistance. The oval-cylindrical housing provides self-stability and high sensitivity, as shown in Figure 9a. In a marine environment with a large sloshing amplitude, the self-stability of the elliptical cylinder is greatly advantageous [64]. Wang et al. investigated a method that can be used to obtain energy from water and can also be used to actively detect the position and velocity of flowing water based on the output power of the generator, as shown in Figure 9b [65]. Columnar structures are widely used in the design of TENGs due to their inherent stability. The cylindrical structure can achieve horizontal flipping and rolling motion and has good balance, fast reset speed, and good robustness. In recent years, the performance of DC output wave energy acquisition device based on cylindrical structure has been summarized in Table 6.

#### 2.2.3. Pendulum Structure Energy Harvesting Device

**Figure 10:**
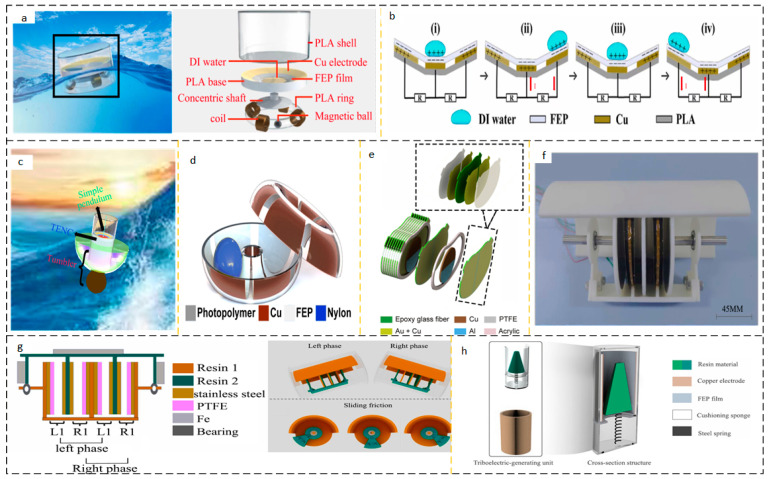
(**a**) iTEHG working scenario and structure, (**b**) working principle. (i) In the initial state, DI water is in contact with the bottom FEP, and the electrode has no charge; (ii) the movement of the device makes DI water move to the right FEP, breaking the original charge balance, and the charge is transported to the middle electrode through the external charge electrode; (iii) DI water returns to its original position, the original charge balance between DI water and FEP is realized, and the charge of the middle electrode is small; (iv) similarly, the charge is transported to the middle electrode again. Reprinted with permission from Ref. [67]. Copyright 2023, the Author(s). Published by Elsevier Ltd. (**c**) AR-TENG typical pendulum working structure. Reprinted with permission from Ref. [68]. Copyright 2021 Elsevier Inc. (**d**) TS-TENG structure diagram. Reprinted with permission from Ref. [69]. Copyright 2019, Elsevier Ltd. (**e**) Schematic diagram of PS-TENG structure. Reprinted with permission from Ref. [70]. Copyright, 2019 Elsevier Ltd. (**f**,**g**) SC-TENG outline diagram and structure, motion schematic diagram. Reprinted with permission from Ref. [71]. Copyright, 2022 Elsevier Ltd. (**h**) Structure diagram of SSR-TENG. Reprinted with permission from Ref. [72]. Copyright, 2023 Wiley-VCH GmbH.

The potential energy of the mechanical structure is disposed of by the elastic potential energy used by the spring, and the gravitational potential energy can also be collected and utilized. Using the high entropy of the movement of the mechanism, the mechanism collects a certain amount of energy and continuously releases it, and the pendulum structure comes into being. Research has always been needed to develop communication systems in the ocean, and the Arctic Ocean presents particular difficulties for satellite communication power supply. June et al. have studied an Arctic-TENG to power satellite communication systems in the Arctic Ocean. Arctic-TENG is designed to serve low-temperature environments and has inferior electrical performance at room temperature to low temperatures. Using an extra-underwater wave simulator at 0.2 Hz, the peak power density of the Arctic-TENG reaches 21.4 W/m^3^, and one Arctic-TENG can transmit 540 bytes of data per day for one year [66]. Xu et al. investigated an isotropic triboelectric-electromagnetic-hybrid nanogenerator (iTEHG) based on a guide liquid, as shown in Figure 10a.

Its structural design integrates TENG and EMG. The inclined dish panel is inlaid with unique concentric circular electrode pairs that guide the movement of the liquid by gravity, as shown in Figure 10b. The solid–liquid coupling of iTENG as the friction working interface prolongs the service life of iTENG [67]. The tumbler-style pendulum design collects waves and is insensitive to the direction of the incident wave. In order to obtain omnidirectional wave energy, Zhang et al. proposed an active resonance triboelectric nanogenerator (AR-TENG) system made of a single pendulum, tumbler, and flexible ring to optimize the super resonant performance, as shown in Figure 10c. The design of active resonance can reduce the wave frequency threshold while increasing the natural frequency output, improving the energy harvesting efficiency [68]. Liu et al. proposed a toroidal structure TENG (TS-TENG). Similarly, TS-TENG is insensitive to wave directivity and can collect wave energy at different angles of incidence, as shown in Figure 10d [69].

Zhong et al. proposed a pendulum structure PS-TENG, as shown in Figure 10e. The charge output density of TS-TENG can exceed the output of the spherical-shell TENG structure by 13.2 times, reaching 4622 μm^3^. In low-frequency water waves below 0.5 Hz, a peak power density of 14.71 W/m^3^ and an average power density of 31.05 W/m^3^ were obtained [70]. In order to reduce frictional losses, Li et al. designed a synchronous contact separation triboelectric nanogenerator (SC-Teng) with intermittent sliding frictional self-excitation, as shown in Figure 10f. The structure is shown in Figure 10g, and the self-charge can be supplemented by self-rotation, providing the highest possible charge density at the friction-coupled interface moment in the contact separation mode [71]. As shown in Figure 10h, Yang et al. proposed a swinging self-regulating triboelectric nanogenerator (SSR-Teng), which can make it possible to use the broken low-frequency wave energy by adjusting the oscillation frequency and resonance effect. As shown on the right side of Figure 10, the friction coupling interface of the SSR-TENG designed by Yang et al. is designed in such a way that the cone surface of the table is coupled with the inner surface of the peripheral cylinder to form a contact-separation mode. The round table is fastened to the base by a spring, and the center of gravity of the round table is adjusted. The center of gravity and spring selection affect the energy recovery performance of the SSR-TENG. SSR-Teng outputs a peak power of 0.14 mW over a wave height range of 6–11 cm [72]. A feasible solution for improving the output performance is imitating the design concept of the initial pendulum model, including increasing the number of pendulums and adding magnets and coils. Increasing the number of pendulums staggered the initial motion phase of the pendulum and prolonged its motion time. By adding magnets and coils, the activation of charge transfer can be achieved by utilizing the principle of magnetic induction lines being cut. In recent years, the performance of some energy harvesting devices based on pendulum structures has been summarized in Table 7 below.

#### 2.2.4. Bionic Structure Energy Harvesting Device

**Figure 11:**
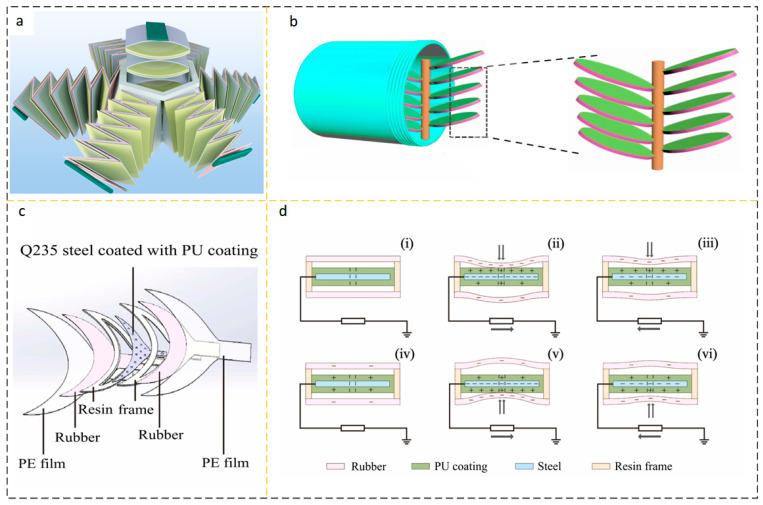
(**a**) FL-TENG shape and structure diagram. Reprinted with permission from Ref. [73]. Copyright 2021, Elsevier Ltd. (**b**) kelp-forest-like TENG seaweed-like TENG structure. Reprinted with permission from Ref. [74]. Copyright 2018, Elsevier Ltd. (**c**) SBF-TENG structure diagram and (**d**) working principle. (i) Initial state; (ii) Squeeze occurs and charges are generated on the rubber; (iii) Release the squeeze, reduce the charge on the rubber, and reverse the direction of the current; (iv) Squeeze the lower rubber, gradually completing charge accumulation on its surface; (v) Squeeze to the maximum limit, and charge accumulation is completed; (vi) Release the squeeze and restore the device to its original charge balance state. Reprinted with permission from Ref. [75]. Copyright 2023, Elsevier Ltd.

One of the treasures that God has given to mankind is life, and human beings continue to explore. Biomimicry has injected life into the development of science. Inspired by the lotus petal opening and closing movement, a FL-TENG consisting of flower-petal sub-TENGs and flower-core sub-TENGs was proposed by Wen, as shown in Figure 11a. The FL-TENG periphery consists of six flower-petal sub-TENGs and flower-core sub-TENGs at the center of the flower, all of which work in contact-separation mode. When the water wave is excited, FL-TENG produces folding and telescopic motions, and the “flowering” and “folding” movements convert kinetic energy into electrical energy. The six flower-petal sub-TENGs mainly collect the kinetic energy of horizontal motion with two degrees of freedom and the kinetic energy of rotational motion with three degrees of freedom, while the core of core sub-TENGs collects the translational kinetic energy in the vertical direction of one degree of freedom [73]. Inspired by kelp that drifts with the waves, Wang et al. studied a kind of TENG (kelp-forest-like TENG) that mimics the movement of kelp and its structure is shown in Figure 11b. At a wave oscillation frequency of 1 Hz, a single kelp-forest-like TNG cell can deliver an *Isc* of 10 μA and a *Voc* of 260 V, which is sufficient to drive at least 60 LEDs with a power density of 25 μW/cm^2^ [74]. As shown in Figure 11c, Zhang et al. first proposed a sealed bionic fishtail structure TENG (SBF-TENG) based on anticorrosive coatings. The SBF-TENG generates an electric potential through the contact separation mode of the rubber layer with the PU coating, as shown in Figure 11d. Both the rubber layer and PU coating have good sealing, stability, and fatigue resistance. SBF-TENG can also be used as a sensitive solution or seawater salinity sensor, and the charge provided by SBF-TENG can be used to build a cathodic protection system and improve the lifetime of metals in the ocean or in solution [75]. In recent years, the performance of some energy harvesting devices based on biomimetic structures has been summarized in Table 8.

## 3. Optimization of Fluid Energy Harvesting Devices

Since the introduction of triboelectric nanogenerators in 2012, the pace of development has been impressive. Compared with solenoid valve click EMG, triboelectric nanogenerators exhibit natural adaptability in low-frequency energy harvesting [76]. After more than a decade of development, the materials used in nanogenerators are safer and more stable and have shown unprecedented biocompatibility, such as when placing them in the human body for cardiovascular sensing and detection [77,78], with high energy density, low space occupancy [79], extremely tolerant environment [80], simple preparation process [81], and low cost. However, triboelectric nanogenerators still need to be improved, and their application occasions and frequencies are still restricted. For example, the power output and energy density of the energy harvesting device are not satisfactory, and the internal structure of the nanogenerator is worn. Obviously, improving the performance of nanogenerators has a positive effect on their use scenarios, use frequency, and commercialization. Recently, research strategies for improving triboelectric nanogenerators have been proposed.

### 3.1. Material and Structural Optimization

**Figure 12:**
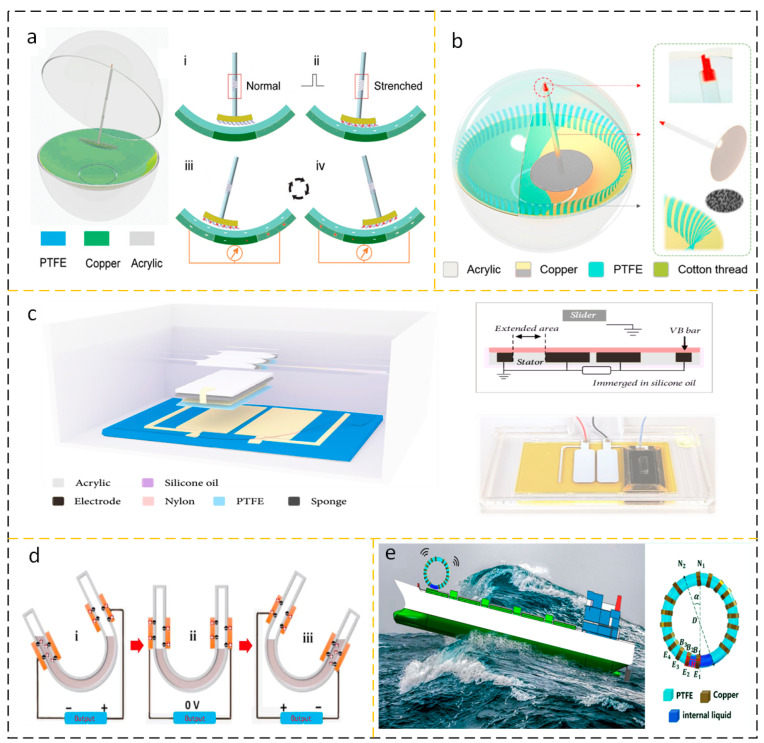
(**a**,**b**) Pendulum TENG with non-contact structure. Among them, (i) is the initial state, and the system has no charge transfer; (ii) is stimulated by vibration, causing the brush to start shaking; When reaching position (iii), PTFE accumulates a large amount of negative charges; Similarly, when reaching position (iv), the brush swings in the opposite direction, producing the opposite current. Reprinted with permission from Ref. [86]. Copyright, 2021 Wiley-VCH GmbH. Reprinted with permission from Ref. [87]. Copyright, 2019 Elsevier Ltd. (**c**) TENG with self-lubricating properties at the friction working interface. On the left is a schematic diagram of TENG with self-lubricating, the upper right image shows the components of TENG with self-lubricating, and the lower right image shows the physical image of TENG with self-lubricating. Reprinted with permission from Ref. [88]. Copyright, 2022 Wencong He et al. (**d**) Schematic diagram of U-shaped TENG. (i) When the U-shaped tube is in the initial position shown in the diagram, there is an equal amount of positive charge on the copper electrode tube wall where the liquid level reaches; (ii) When the U-shaped tube is horizontal, the liquid level does not reach the copper electrode, and there is no charge transport in the external circuit; Upon reaching position (iii), similarly, the liquid near the copper electrode once again accumulates charges due to differences in electronegativity and is transported from the external circuit. Reprinted with permission from Ref. [89]. Copyright, 2018 Tsinghua University Press and Springer–Verlag GmbH Germany, part of ger Nature. (**e**) Cyclical liquid-structure interaction TENG. Reprinted with permission from Ref. [90]. Copyright, 2020 Elsevier B.V. All rights reserved.

The application environment of fluid energy collection devices based on triboelectric nanogenerators is often in deserts or oceans [82], which means that the frequency of manual maintenance cannot be too high, and high-frequency maintenance will bring high labor costs. The presence of mechanical wear may significantly reduce the service life of an energy harvesting device, so choosing better materials is the lifeblood of an energy harvesting device. Better materials need to be more wear-resistant, referring to the triboelectric sequence [83], to be able to produce long-lasting, high-energy output power. The stability and corrosion resistance of inorganic materials are due to organic materials. In 2023, Zhao et al. studied the contact polarization effect between metal electrodes and inorganic materials and found that the electron transfer mechanism is the basis of the charge transfer mechanism for contact charging. The work function of the dielectric layer and atomic species on the metal has an important influence on the charge transfer process when contacting the metal. The friction layer is made of inorganic single crystals, which greatly expands the application of inorganic materials in fluid energy harvesting devices [84]. Han et al. discovered that rabbit hair has the function of reducing frictional resistance, and introduced rabbit hair to the improvement of fluid energy collection devices to design SCR-TENG. When rabbit fur and FEP work together, rabbit fur not only plays a role in reducing friction but also provides more frictional charges and enhances the output power of the fluid energy collection device [85]. The existence of friction pairs that play a non-positive role will definitely shorten the life of the mechanical structure. The friction material is made into a brush shape, which greatly reduces the wear between materials. A dual-mode and frequency-doubled TENG with ultra-high durability and efficiency through elastic connection and soft contact design is used for ultra-low frequency mechanical energy harvesting. By introducing springs and flexible dielectric fluff into a novel pendulum structural design, the TENG’s surface tribocharge is replenished in a soft contact mode under intermittent mechanical excitation, while the robustness and durability are enhanced in a non-contact operating mode, as shown in Figure 12a,b [86,87].

Similarly, the use of liquid lubrication effectively reduces the wear between friction media. He et al. proposed a liquid lubrication-facilitated TENG, namely LP-TENG. The reasonable design of the voltage balance rod can obtain an energy output density of 87.26 W/m^2^ under the low-frequency disturbance of 2 Hz by using the space charge accumulation effect. This energy output density makes the output power of LP-TENG comparable to that of solar panels, as shown in Figure 12c [88]. The use of pendulum structures, flutter structures, etc., is all prepared to reduce friction pairs. However, another way to reduce frictional losses is to adopt a structural model of solid–liquid contact and solid–gas contact. At present, the most studied mode is solid–liquid contact, and its general structure is to use a solid container of a specific shape, such as an optional FEP, which encapsulates special liquid and metal electrodes according to the topological shape of the container.

Pan et al. had a Voc of 81.7 V and an Isc of 0.26 μA for pure water-based U-tube TENG at 0.5 Hz shaking. The improved sandwich-like U-tube TENG exhibits excellent output performance with a Voc of 350 V, an Isc of 1.75 μA, and a power density of 2.04 W/m^3^, as shown in Figure 12d [89]. Wang et al. made a TENG tilt sensor. A ring-shaped PTFE tube is used, a certain amount of water is filled inside, and when a certain inclination angle is given, the specific charged contact mechanism between PTFE and water forms the movement of charge, generating electric potential and current. It is proven that there is a proportional relationship between the output voltage and current and the tilt angle of the TENG tilt sensor. This packaged self-powered sensor device is particularly suitable for the operating environment of ocean ships, as shown in Figure 12e [90]. 

### 3.2. Expending Hybrid Energy Harvesting Device

**Figure 13:**
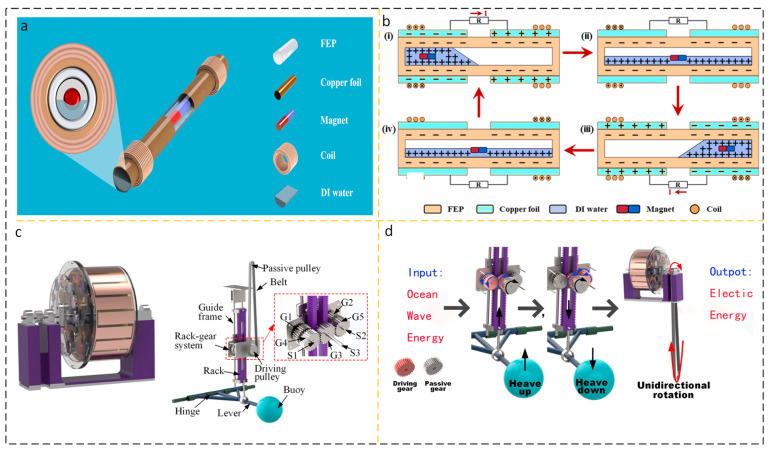
(**a**) Solid–liquid-electromagnetic coupling TTENG and (**b**) working principle. (i–iv) From the initial tilt to the horizontal, the affinity of DI water for negative charges induces charge, and the addition of magnets can also enhance power generation efficiency; Similarly, the movement of a magnet undergoes Faraday electromagnetic induction wit h the surrounding coils. Reprinted with permission from Ref. [92]. Copyright, 2022 Elsevier Ltd. (**c**) Composite wave energy harvesting element (WEC) integrating mechanical transmission mechanism, float, TENG, and electromagnetic induction generator, and (**d**) tooth ratio velocity amplification mechanism. Reprinted with permission from Ref. [93]. Copyright, 2021 Elsevier Ltd.

The power generated by wave energy fluid energy harvesting devices (WEHs) depends on the fluid disturbance in the current working environment, which means there is a strong correlation between the disturbance and the output power. Under weak disturbances, the output power is naturally insufficient, and the volume of a single TENG is limited. The contact area of the frictional layer is limited, which makes it difficult to meet people’s demand for electricity for a long time due to the transfer of charges. At present, the output power of a single TENG is limited, and most of the output power is concentrated in the mW order of magnitude. Parallel multi-group TENG is a solution that has been practiced for a long time. After networking, TENG effectively concentrates the energy of multiple individual devices through reasonable design of control circuits, achieving high-capacity output capacity. However, fundamentally, improving individual TENG is the fundamental guarantee for solving the problem of high-capacity TENG networking. A feasible solution to improve the efficiency of a single TENG is to integrate TENG with ENG to form a hybrid energy harvesting system. The working frequency of electromagnetic generator EMG is higher, and compared to TENG, it has the characteristics of high current and low voltage. TENG itself has a higher output voltage, but a lower current. The two work in different working environments and can complement each other’s shortcomings [91,92]. Sun et al. studied a liquid-solid electromagnetic combined water kinetic energy harvesting nanogenerator (TTENG). In addition to the traditional structure mentioned above, an innovative approach is to add a permanent magnet and an induction coil to combine TENG with EMG, resulting in consistent current direction and significantly enhancing the output level of TENG, as shown in Figure 13a,b [93]. Zhao et al. designed a composite wave energy harvesting element (WEC) consisting of a mechanical transmission mechanism, a floating ball, TENG, and an electromagnetic induction generator. In this system, the power generation system is designed in a cylindrical shape. The side of the cylinder is equipped with permanent magnets and coils, and it is an electromagnetic induction generator RD-EMG. The cylindrical surface of the cylinder is equipped with MBC-TENG, and the friction layer material is selected as PTFE and nylon brush. The floating ball receives wave disturbance in the water, and its motion is transmitted to the floating ball. The speed is then increased through gear racks and a carefully proportioned variable speed gear mechanism, and the rotational kinetic energy is transmitted to MBC-TENG and RD-EMG, as shown in Figure 13c,d [94]. It has been proven that based on the difference in working frequency, ENGs and TENGs working together can greatly improve the efficiency of energy harvesting equipment.

### 3.3. Development of High-Power TENG

At present, a large number of studies have focused on the output electrical performance of TENG, extending the service life, and reducing the threshold of external excitation energy, and the results of their research are impressive. However, as mentioned above, due to some current limitations, the electrical output of TENG is still some way from practical use. Therefore, combining existing advanced technologies, such as semiconductor technology, into TENG will greatly increase the output power of TENG, broaden the research prospects of TENG, attract more researchers to join TENG research, and create a virtuous circle in the field of TENG and its interdisciplinary fields such as triboelectronics [95].

**Figure 14:**
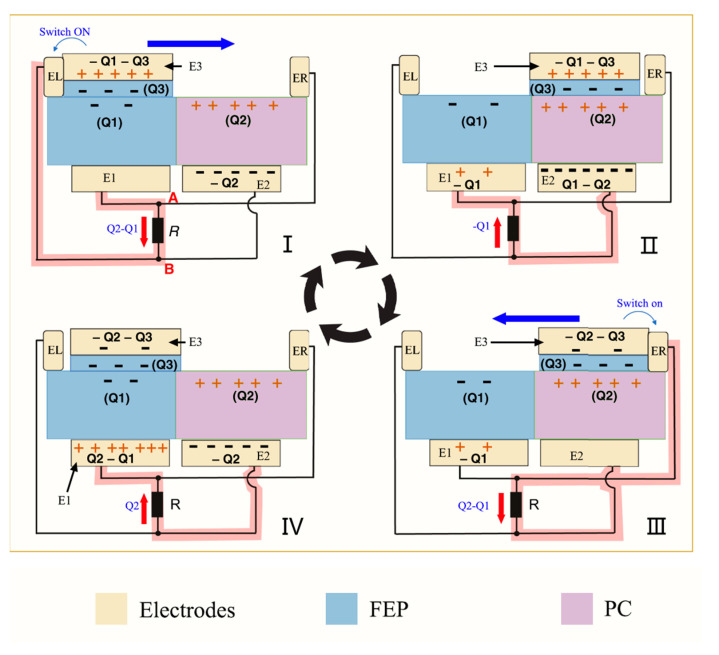
OCT-TENG working principle diagram. (**I**) The source E3 slider is in contact with the left gate, and the gate EL is conducting; (**II**) The source E3 slider is separated from the gate (gate) EL, free between the two gate EL and ER without contact, and the two gate EL and ER are turned off; (**III**) The source E3 slider is in contact with the right gate, and the gate ER is conducting. At this moment, the left half is considered insulated and there is no change in charge; (**IV**) The source E3 slider is separated from the gate (gate) ER, free between the two gate EL and ER without contact, and the two gate EL and ER are turned off. Reprinted with permission from Ref. [96]. Copyright, 2021 the Authors. Published by Springer Nature.

Combined with transistor technology, Wu et al. innovatively proposed a high-performance and high-power OCT-TENG. As shown in Figure 14, the core of OCT-TENG is the use of reverse charge enhancement and transistor-like designs to increase the charge output and reduce the output impedance. The OCT-TENG consists of a stator substrate and a slider. The stator contains a coplanar friction surface with opposite charge polarization (fluorinated ethylene propylene, abbreviated as FEP, and polycarbonate, abbreviated as PC, both 100 μm thick) and four electrodes (E1, E2, EL, and ER). EL and ER are two floating electrodes placed on the left and right sides of the stator substrate. E1 and E2 are two sheet electrodes (Cu) placed below the FEP and PC membranes. The slide contains the sheet electrode E3 below the FEP film (10 μm). The entire device resembles a pair of complementary transistors and consists of two transistor-like components with the same structure and opposite friction surfaces. E1 and E2 can be thought of as the “source” (S) of each “transistor”, and E3 can be thought of as a dynamic “drain” (D). EL and ER are the “gates” (G). When E3 comes into contact with EL or ER, the transistor’s “gate” is switched to “on”, so that charge can flow between the “source” and “drain” [96].

## 4. Summary and Outlook

Fluid energy is a vast, mostly untapped source of power for the human species. The formation of wave energy from wind energy and currents is not only common in our everyday lives but also contains large reserves. With the advent of the information age, the demand for electricity as an energy supply undoubtedly increased. The gap between the demand and supply of energy is becoming larger and deeper. The proliferation of sensors and data-transfer infrastructures will lead to steadily rising energy demands. This indicates that we need to think about and be accountable for the preservation of the natural environment [97]. Second, sensors are becoming more pervasive in the Internet of Things age. Distributed sensor networks, also known as intelligent sensing networks, are set up in the manner and location determined by human needs. As a result, there will be a growing need for decentralized sources of energy. New energy sources such as various batteries and solar energy may not necessarily meet such large capacity needs. The age of big data and the Internet of Things is here, and the triboelectric nanogenerator is a revolutionary technology that enables the use of a decentralized energy supply for components. Simultaneously, it marks a new turning point in the evolution of the electrification and information age and hastens the advent of the micro-electromechanical era. The self-powered capability of nanogenerators may significantly increase their range of applications, including intelligent sensing systems, the collection of wave and wind energy, biological and medical health and monitoring, pollution prevention, and control, etc. This system includes a nanogenerator, a converter, and a storage device. For the nanogenerator system, the energy receiver is the first point of energy input. The collection of wind energy is more likely to produce high-frequency oscillations than either water kinetic energy or biological energy. There are primarily flutter structures and rotating structures because films are often employed to enhance the friction frequency of the friction layer. It is important to capture instantaneous kinetic energy, transform it into potential energy, and then transfer the energy via a transducer since the chance of capturing water kinetic energy is large at low frequencies. Vibration energy is undeniably a vast repository of treasure, despite the fact that its effective collection is currently impossible. The existing energy difficulties may be solved in part by gathering enough vibration energy with a suitable energy conversion rate and reserve [98].

The decentralized energy supply additionally presents prospects for the advancement of real-time sensor network monitoring. For example, in desert hinterlands or vast oceans, the era of electrification means that only turbine wind turbines and solar converters are used without a stable self-energy supply. Conversely, nanogenerators provide a less expensive and less prone to failure alternative for the power supply of warning systems installed on buoys and climate monitoring devices in the desert. This article starts with the nanogenerator material that requires two or more friction layers with high electronegativity to create the induced electromotive force acquired by contact and electrostatic induction in response to a mechanical movement. This section introduces the triboelectric sequence that is necessary for nanogenerators. Generally speaking, choosing friction materials necessitates choosing two or more materials in the sequence in which it is simple to gain electrons and easy to lose electrons in order to offer the highest feasible energy conversion rate. Second, the fundamental theory behind the nanogenerator—the second term of Maxwell’s displacement current that has been “ignored” for a long time is briefly presented and its working principle is examined. This paper introduces the four basic modes that are currently commonly used in nanogenerators: single electrode type, independent layer type, vertical contact separation type, and horizontal sliding type. To present the research development of nanogenerators in the area of fluid energy collection, energy collection can be separated into wind energy and water flow wave energy based on the collected fluid medium and the structural design of the generators. The advancements in fluid energy harvesting-based nanogenerator research highlight some issues and provide remedies from earlier studies.

At the moment, the investigation of a significant number of nanogenerators is plagued by a number of flaws that need to be fixed. Firstly, the coupling structure of gas flow and coupling devices or wave and coupling devices is the primary focus of current energy harvesting research in nanogenerators. Though a lot of progress has been achieved in the mechanical structure, research on semiconductors and triboelectric materials is still insufficient. Especially, after multiple wear and tear, the mechanical tensile strength and mechanical strength may suffer from attenuation [99]. Second, although one of the development trends is to work with the network to create a large-scale network for nanogenerator power generation, the energy reserve and power management systems of nanogenerators have not yet reached a very mature state following the networking. This is because the power generation power of nanogenerators is currently relatively low. The coordination of the power generation network and the circuit must be studied when TENG constructs a power generation network or adopts an excitation circuit. Not only would improper use of excitation and power management circuits fail to optimize the efficiency of each TENG, but it may also result in a decrease in the total power output. As a result, research on TENG network excitation and power management circuits is also ongoing. Thirdly, in order to attract more researchers to the topic, equations or theories pertaining to the dynamics of nanogenerators should be substantially simplified. Ultimately, the development of nanogenerators requires dependability. In the TENG network that collects ocean wave energy, the transmission line should have sufficient wind and wave resistance, the link between the transmission line and the TENG should have fatigue tolerance, and the power attenuation of long-distance transmission should be carefully checked. The development of nanogenerators is motivated by the fact that they are maintenance-free after a specific design life, and if they are not dependable enough and need human maintenance, the value of nanogenerators will not be completely embodied. Thus, in the realm of semiconductors, the design of ordinary semiconductor devices might be compared to the design of nanogenerators for energy harvesting.

Transistor principles inspired the fabrication of OCT-TENG, which has triboelectric enhancement with reverse charge enhancement. The porosity and pore depth of the functional rubber film can be controlled. Using transfer printing to manufacture micropores, periodic porous morphology can be achieved on the surface of rubber molds while maintaining the typical mechanical strength and super stretchability of rubber. Coupled 3D printing is also a new research hotspot. The coupled 3D printing transfer printing technology developed by researchers can produce double-layer frictional PDMS films, which can provide better charge transfer efficiency [100]. A breakthrough in nanogenerator friction layer material allows designers to choose materials with low maintenance costs, low raw material costs, excellent manufacturability, environmental friendliness, and high power generation performance, greatly expanding nanogenerator use scenarios. The concept of TENG networking to produce electricity and create a power-generating array was put out by Wang et al. in 2017 [101,102]. The use cases of TENG may be increased by arranging the grid in a sensible way [103]. Thirdly, an essential solution to the energy storage issues of nanogenerator power-generating networks may be the development of high-performance asymmetric supercapacitors (ASCs), hybrid supercapacitors (HSCs), and hybrid energy storage devices (BSHs) comprising supercapacitors and lithium-ion batteries. Supercapacitors are low-energy-density devices with high power densities. Lithium batteries, on the other hand, have a high energy density but a low power density; the two together will effectively address the technical issues with energy release and storage [104,105,106,107,108,109,110]. Eventually, the key to repairing mechanical damage is to develop materials that are highly wear-resistant, self-lubricating, and self-healing. However, the best way to address wear and friction is to develop solid–liquid TENG that does not have an interaction structure or mutual corrosion.

## Figures and Tables

**Table 1:** Performance of wind energy harvesting devices based on rotating structure AC output in recent years.

TENG	Operating Wind Speed Range	Short-Circuit Current (μA)	Open-Circuit Voltage	Power (mW)	Ref.
RS-HG	5~25 m/s	8.74	1.44 kV	7.85	[31]
D-TENG	2.2~16 m/s	18.9	228 V	2.0	[32]
Hybrid generator	9 m/s	14.6	190 V	0.33	[33]
WM-TENG	1.6~6.7 m/s	168	6.8 V	None	[34]
MM-TENG	0~6 m/s	7.9	490 V	6.55	[35]

**Table 2:** Performance of wind energy harvesting devices based on rotating structure DC output in recent years.

TENG	Operating Wind Speed Range	Short-Circuit Current	Open-Circuit Voltage	Power/Power Density	Ref.
TENG-EMG	3.5~6 m/s	6 mA	600 V	0.7 W	[36]
WH-EH	8~9 m/s	1.48 μA	204 V	${195.2\;mW/m}^{2}$	[37]
WNG	1.65 m/s	125 nA	250 V	0.753 μW	[38]

**Table 3:** Performance of self-regulating wind energy harvesting devices based on rotating structures in recent years.

TENG	Operating Wind Speed Range	Short-Circuit Current	Open-Circuit Voltage	Power/Power Density	Ref.
SA-TENG	5~13.2 m/s	14 μA	500 V	7.69 mW	[39]
SM-TENG	2~25 m/s	16 μA	1100 V	3.47 mW	[40]

**Table 5:** Performance of AC output wave energy collection devices based on cylindrical structure in recent years.

TENG	Operating Frequency	Short-Circuit Current	Open-Circuit Voltage	Power/Power Density	Ref.
WS-TENG	5–57 Hz	17.04 μA	116 V	0.0626 mW	[60]
WLM-TENG	1 Hz	30 μA	3 kV	50 mW	[61]
multifunctional TENG	2 Hz	24 μA	490 V	15 mW	[62]
FMC-TENG	0.33 Hz	7.5 μA	400 V	$6.67{\;mW/m}^{3}$	[63]

**Table 6:** Performance of DC output wave energy collection devices based on cylindrical structure in recent years.

TENG	Operating Frequency	Short-Circuit Current	Open-Circuit Voltage	Power	Ref.
EC-TENG	0.25~25 Hz	2 μA	63 V	90 μW	[64]
TENG			4 V	0.9 μW	[65]

**Table 7:** Performance of energy harvesting devices based on pendulum structure in recent years.

TENG	Operating Frequency	Short-Circuit Current	Open-Circuit Voltage	Power/Power Density	Ref.
Arctic-TENG	0.2 Hz	32 μA	1000 V	$21.4{\;W/m}^{3}$	[66]
iTEHG	0.67~1.47 Hz	2 μA	200 V	$7.25{\;\mu W/cm}^{3}$	[67]
AR-TENG		125 μA	70 V	12.5 mW	[68]
TS-TENG	2 Hz	0.3 μA	84 V	$0.21\;{mW/m}^{2}$	[69]
PS-TENG	0.5 Hz	2.77 μA	3072 V	$14.71{\;W/m}^{3}$	[70]
SC-TENG	1 Hz	16 μA	1284 V	8.3 mW	[71]
SSR-TENG	1 Hz	10 μA	120 V	0.14 mW	[72]

**Table 8:** Performance of energy harvesting devices based on bionic structure in recent years.

TENG	Operating Frequency	Short-Circuit Current	Open-Circuit Voltage	Power/Power Density	Ref.
FL-TENG	0.9 Hz	48 μA	160 V		[73]
kelp-forest-like TENG	1 Hz	10 μA	260 V	$25\;{\mu W/cm}^{2}$	[74]
SBF-TENG	2 Hz		7.21 V	$1.67\;{mW/cm}^{2}$	[75]

## Data Availability

Not applicable.
